# Development of peptoid-based ligands for the removal of cadmium from biological media†
†Electronic supplementary information (ESI) available. See DOI: 10.1039/c5sc00676g


**DOI:** 10.1039/c5sc00676g

**Published:** 2015-05-14

**Authors:** Abigail S. Knight, Effie Y. Zhou, Matthew B. Francis

**Affiliations:** a Department of Chemistry , University of California , Berkeley , CA 94720 , USA . Email: mbfrancis@berkeley.edu; b The Molecular Foundry at Lawrence Berkeley National Laboratory , Berkeley , CA 94720 , USA

## Abstract

Cadmium poisoning poses a serious health concern due to cadmium's increasing industrial use, yet there is currently no recommended treatment. The selective coordination of cadmium in a biological environment—*i.e.* in the presence of serum ions, small molecules, and proteins—is a difficult task. To address this challenge, a combinatorial library of peptoid-based ligands has been evaluated to identify structures that selectively bind to cadmium in human serum with minimal chelation of essential metal ions. Eighteen unique ligands were identified in this screening procedure, and the binding affinity of each was measured using metal titrations monitored by UV-vis spectroscopy. To evaluate the significance of each chelating moiety, sequence rearrangements and substitutions were examined. Analysis of a metal–ligand complex by NMR spectroscopy highlighted the importance of particular residues. Depletion experiments were performed in serum mimetics and human serum with exogenously added cadmium. These depletion experiments were used to compare and demonstrate the ability of these peptoids to remove cadmium from blood-like mixtures. In one of these depletion experiments, the peptoid sequence was able to deplete the cadmium to a level comparable to the reported acute toxicity limit. Evaluation of the metal selectivity in buffered solution and in human serum was performed to verify minimal off-target binding. These studies highlight a screening platform for the identification of metal–ligands that are capable of binding in a complex environment. They additionally demonstrate the potential utility of biologically-compatible ligands for the treatment of heavy metal poisoning.

## Abstract 2

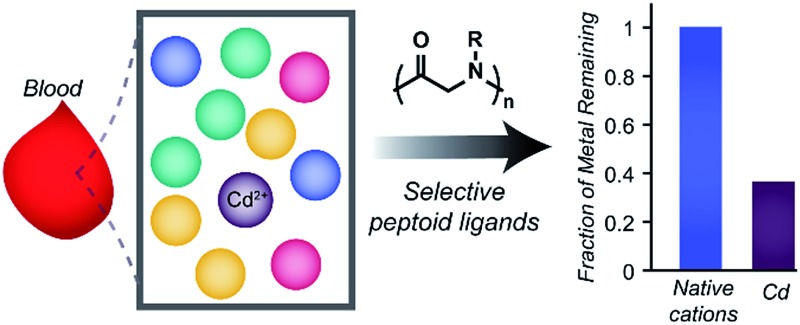
To address the lack of current therapeutic strategies for cadmium poisoning, peptoid-based ligands are identified using combinatorial chemistry that can selectively coordinate cadmium in a complex biological sample matrix.

## Introduction

For the past fifty years it has been well established that cadmium is hazardous to human health, but the industrial use of this metal has continued to increase world-wide.^1^ Cadmium exposure leads to a particularly problematic form of heavy metal poisoning due to the lack of current treatment options.^2^ Cadmium has been identified by the International Agency for Cancer as a group-1 carcinogen, and it is listed by the Environmental Protection Agency as one of 126 priority pollutants.^3^ Despite this toxicity being well accepted, it is not fully understood how cadmium interacts with biological molecules to produce these deleterious effects.^4^ One current hypothesis suggests that Cd^2+^ can interfere with DNA repair and protein function, and that it can mediate the production of reactive oxygen species despite not acting directly as an oxidant.^4^–^6^ Two biological molecules, metallothionein proteins and glutathione, can aid in the clearance of Cd^2+^. Unfortunately, metallothioneins can increase the circulation time of cadmium ions,^7^ and the glutathione depletion can have debilitating effects on cells, potentially including the observed oxidative stress responses.^8^

Evolution does not appear to have provided a strategy for clearing Cd^2+^, and unfortunately there is also no therapeutic solution to cadmium poisoning. Chelation therapy has been proven to be beneficial for some heavy metals including lead and mercury, and has become the mainstay of treatment for acute poisoning with these metals.^9^ Simple small molecules, including EDTA, dimercaptosuccininc acid, and 2,3-dimercaprol, are commonly used for these purposes. One major concern with the administration of chelation therapy is that despite the increased affinity of many small molecule chelators for their heavy metal targets, the significant excess of other biological ions makes elements such as Ca^2+^ their primary target. This leads to a variety of hazardous side effects.^10^ There exists a clear need for non-toxic ligands with selective affinity for cadmium that could be introduced as a chelation therapy alternatives. Additionally, such ligands could be integrated into other blood treatment strategies, such as dialysis platforms.

Selective chelation of Cd^2+^ in blood is a considerable challenge due to the significant excess of competing ions and ligands, including serum small molecules and proteins. The rational design of a ligand for this application is particularly difficult due to the chemical similarity between Cd^2+^ and biologically essential Zn^2+^. Peptide-based combinatorial approaches have been demonstrated to successfully identify ligands as catalysts and metal tags,^11^–^14^ and pioneering work on peptide-based synthetic ionophores was completed by W. C. Still.^15^ We propose that peptoids, or *N*-substituted glycine oligomers,^16^ offer distinct advantages for the treatment of cadmium poisoning. The ability of these peptidomimetics to access unique conformations and the variety of available monomers make peptoids particularly valuable as ligands, and their metal binding capacity has been recently demonstrated.^17^–^21^ Additionally, their improved *in vivo* stability provides exceptional advantages for a therapeutic application.^22^–^24^ In this work, we have developed an expanded peptoid-based library and used it to identify multiple ligands with the ability to bind Cd^2+^ in human serum. One of these structures was found to reduce the concentration of Cd^2+^ to a level comparable with the reported limit of acute toxicity.^25^ These studies therefore reinforce the unique capabilities of peptoid-based ligands through the development of structures with unparalleled ion-selectivity in a particularly complex biological medium.

## Results and discussion

### Library design and synthesis

**Fig. 1:**
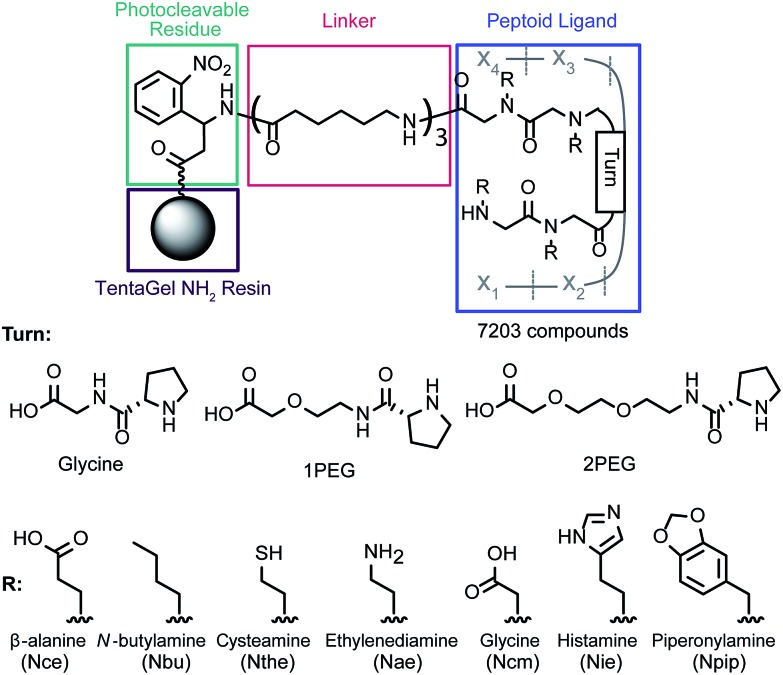
Peptoid library schematic. The diagram depicts the photocleavable residue, linker, and peptoid ligand structure with variable positions represented as “X_*n*_” and “Turn”. The seven “R” residues were incorporated into each of the four peptoid positions, and the three d-proline based turns were incorporated into the “Turn” position. The overall library consists of 7203 compounds.

To approach the challenge of developing a high affinity ligand that can bind to cadmium in the complex medium of human blood, we synthesized a peptoid-based library designed to decrease the number of available conformations and form a binding “pocket.”^26^ In our previous work, a peptoid tetramer was synthesized and demonstrated to have the potential for selective binding; additionally it was discovered that the incorporation of a d-proline-based turn increased the metal affinity of the ligand.^17^ Based on this observation a library incorporating d-proline and three turn moieties (Glycine, 1PEG, and 2PEG) was designed (Fig. 1), with the objective of discovering higher affinity and more selective structures. Within this work we will define selectivity as the preferential binding of cadmium in the presence of biologically relevant concentrations of competing metal ions.

The library members were synthesized on 140–170 μm PEG-grafted polystyrene resin, which is compatible with both organic solvents and water for the synthesis and screening steps, respectively. The first residue incorporated was a photocleavable moiety to allow cleavage and sequence identification using MALDI-TOF MS/MS. This residue and the following three linker groups were added using standard solid-phase peptide chemistry. Previously, a residue with an isotopic tag (^79^Br/^81^Br) was included to identify library member mass signals; however, due to the decreased signal-to-noise ratio we chose not to include this residue for this screen. To increase the mass of the cleaved structures and the distance between the ligands and the resin, three aminohexanoic acid residues were combined to form a linker. The ligand itself was synthesized using a combination of the submonomer method of peptoid synthesis^27^ with chloroacetic acid to allow incorporation of heterocyclic side chains^28^ and standard Fmoc-peptide synthesis. Split-and-pool synthesis was used to create a library of 7203 members. Adducts between the histamine and chloroacetic acid were cleaved with 4-methylpiperidine^28^ and acid labile protecting groups on the monomers were cleaved with trifluoroacetic acid.

### Screening for selective cadmium affinity

**Fig. 2:**
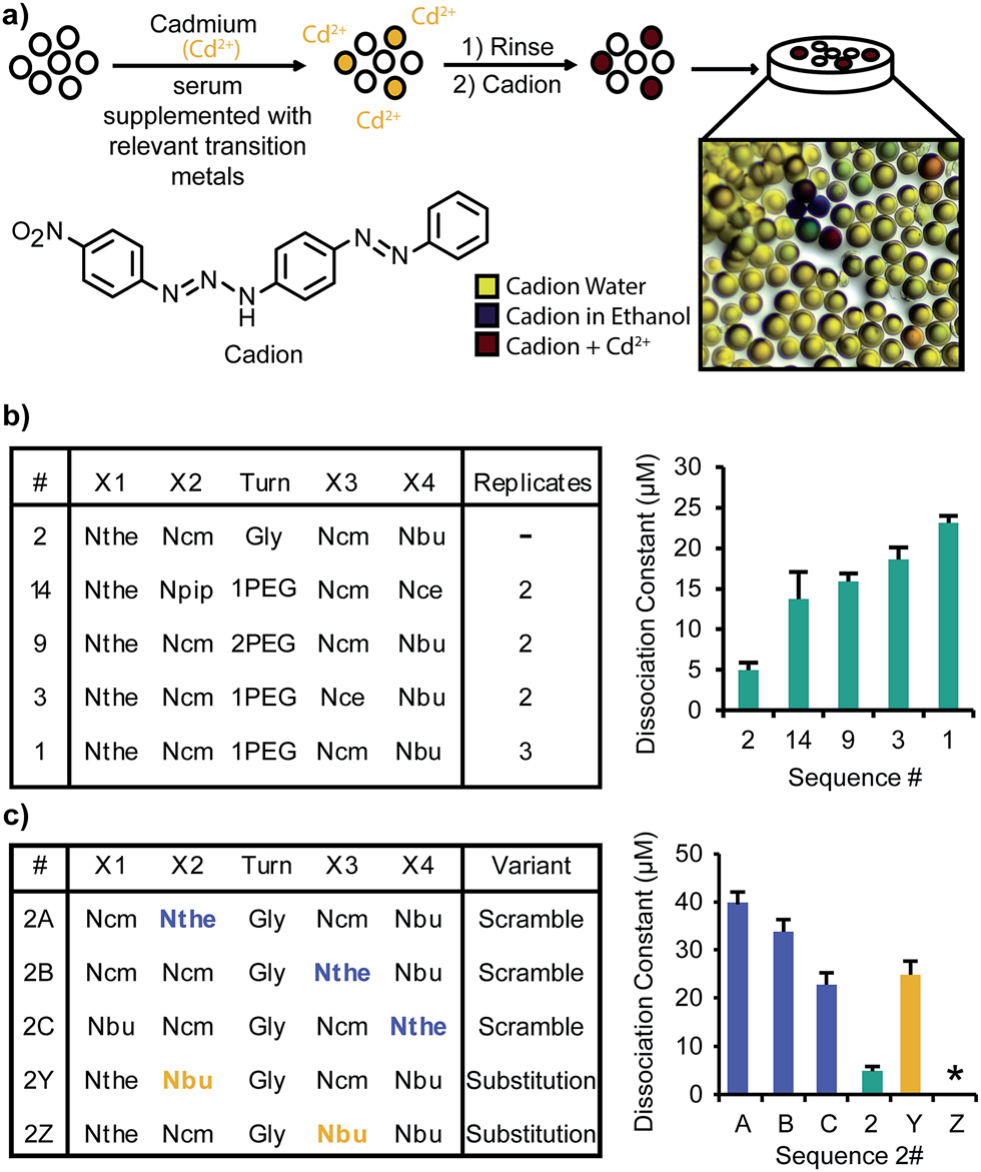
Overview of the screening procedure and measured Cd^2+^ binding affinities of identified sequences and variants. (a) An outline of the screening procedure is shown with a representative photograph of the library members after the staining procedure. The screening medium included serum replacement, added transition metal ions found free in blood (Fe^2+^, Mn^2+^, and Zn^2+^, 250 μM) and cadmium (25 μM). After incubation of the library with the screening medium, the library was rinsed and treated with cadion dye. (b) Five of the eighteen identified sequences are listed. All dissociation constants displayed were measured using titrations in HEPES buffer (10 mM, pH 7) monitored by UV-vis spectroscopy. A logistic fit was applied to the data and the inflection point was used to approximate the *K*_d_ values. A 1 : 1 binding mode was assumed. c) Variants of the highest affinity sequence, Sequence 2, are shown. 2A–C are scrambled sequences in which the thiol containing monomer (Nthe) was switched with each residue. 2Y and 2Z are structures in which the acid containing moieties (Ncm) were replaced with Nbu to investigate the significance of each residue. The binding was characterized using the same method as (b); however, for Z, the affinity was too low to measure. All error bars represent the standard error of the logistic fit.

To identify ligands that could bind cadmium in the presence of the proteins, small molecules, and other ions in human serum, a screening medium that contained reproducible concentrations of these components was designed. A serum replacement, intended as a media supplement for mammalian cell culture, was obtained from Life Technologies. Excess biologically relevant transition metals (Fe^2+^, Mn^2+^, Zn^2+^), typically present in high concentrations or unbound in serum,^29^,^30^ were considered to be the main competing ions. These were added to the serum replacement (2.5 mM each) in addition to Cd^2+^ (250 μM). An outline of the screening procedure is shown in Fig. 2a. A library aliquot pre-swelled in water was exposed to this screening medium for 1–4 h before being isolated *via* filtration and rinsed with water. Because cadmium typically forms colorless complexes, a dye that forms distinctly colored complexes with these ions was applied to assist in the detection of ligands within the library. The dye was applied in ethanol, which after evaporation left the metal and cadion dye^31^,^32^ trapped within the beads. The cadion dye is yellow in water and blue in ethanol, leading to the yellow, blue, and green beads apparent in Fig. 2a. However, a bright pink color occurs upon complexation of cadmium (color with relevant metals shown in Fig. S1a†), allowing unambiguous identification of the cadmium chelators using a light microscope.

The dye and complexed ions were removed from the selected beads to prepare for sequencing using MS/MS (see the ESI† for details). Photocleavage was performed, but as depicted in ESI, Fig. S1,† the photocleavage led to a mass loss of 32 for the identified sequences. It was assumed that this loss corresponded to desulfurization under the photolysis conditions. To verify the mass of the original structure, the screening procedure was repeated and the selected beads were incubated with *N*-ethylmaleimide (NEM) before photocleavage to cap the thiol residues and prevent any undesired photochemical reactions (ESI, Fig. S1d†). This procedure yielded an individual bead with both the NEM capped structure and the uncapped structure. By comparing the MS/MS fragmentation patterns of the two structures, it was clear that the mass of 32 was lost from an N-terminal Nthe residue during the photocleavage step.

After multiple rounds of screening, eighteen unique sequences were identified (full structures are shown in ESI, Fig. S2†). All but two of these structures contained an N-terminal Nthe residue, with the negatively charged residues (Ncm and Nce) common in positions X_2_ and X_3_. Interestingly, the Nthe group was not identified in other positions. There was no consensus for the turn moiety, and many of the unique ligands varied only by that residue. The final position, X_4_, was mostly filled by hydrophobic residues (Nbu and Npip).

### Binding affinity characterization

To compare the affinity of each of the structures for Cd^2+^, titrations of Cd^2+^ into solutions of each peptoid were completed and monitored by UV-vis spectroscopy. The peptoid structures were prepared by synthesis on Rink Amide resin and purified by reverse phase HPLC after cleavage from the resin. Example mass-spectra of sequences after purification are shown in ESI, Fig. S3.† The titrations were monitored at a wavelength of 245 nm. A broad peak spanning from 260 nm through the lowest wavelength scanned (220 nm) was apparent; 245 nm was chosen to avoid wavelengths where the solvent and isolated peptoid would absorb. Absorbance at these low wavelengths is characteristic of cadmium thiolate clusters.^33^ The data sets were fit to logistic curves and the inflection points were used to determine the dissociation constants (ESI, Fig. S4†). The stoichiometry of the complex formed is difficult to determine due to other competing reactions (*e.g.* disulfide formation). The complexes did not remaining intact after MALDI-TOF MS ionization, and thus could not be detected directly. In this work, we assume a 1 : 1 ratio based on previous characterization of similar complexes formed with glutathione at comparable concentrations.^34^ The *K*_d_ for the highest affinity peptoid, Sequence 2, and the highest affinity structures that were identified multiple times in the screen (Sequences 1, 3, 9, and 14) are shown in Fig. 2b. Generally, the *K*_d_ values ranged from 5–40 μM. As a preliminary evaluation of the selectivity of the peptoids, the same titration was performed with Sequence 2 and Zn^2+^, and an eight-fold lower affinity was observed (ESI, Fig. S5c†). To compare the identified sequences to a similar biological compound, a titration was performed with glutathione and cadmium. Glutathione had an affinity too poor to characterize with the titration (ESI, Fig. S5b†).

To understand the mechanism of Cd^2+^ binding and inform any future ligand evolution, the specific interactions of each residue with Cd^2+^ were probed. To begin, the location of the Nthe residue (at the N-terminus) was switched with each of the other residues in the highest affinity ligand (Sequence 2). The structures and measured *K*_d_ values are shown in Fig. 2c as variants 2A, 2B, and 2C. These rearrangements yielded four- to eight-fold decreases in affinity, indicating not only the necessity of the Nthe residue, but also the importance of its location in the ligand. To analyze the importance of each of the two carboxylic acids, variants were synthesized with Nbu residues replacing the Ncm functionalities (variants 2Y and 2Z). Each of these had a significantly decreased affinity, and 2Z (containing Nbu at the X_3_ position) had a *K*_d_ value larger than what could be determined with the titration (ESI, Fig. S5a†).

### Complex characterization with NMR

**Fig. 3:**
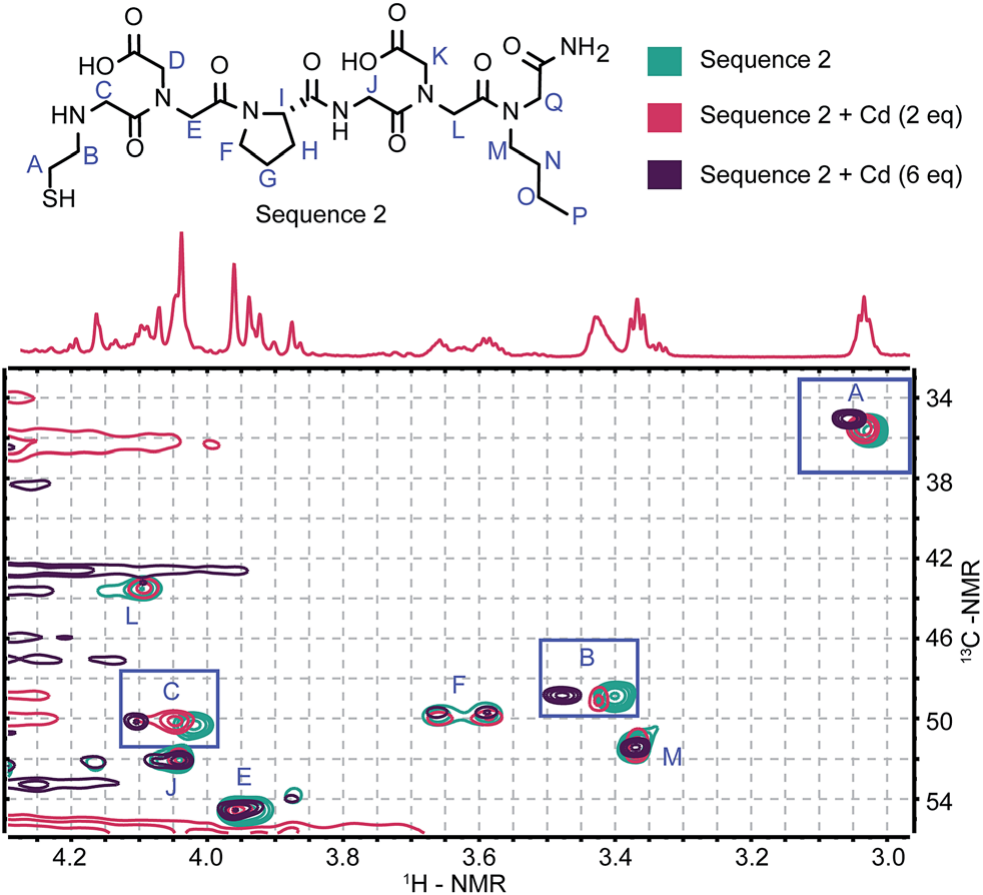
^1^H–^13^C HSQC NMR (900 MHz) spectrum of Sequence 2 (500 μM) in the presence of Cd^2+^. Resonances that shift upon the addition of Cd^2+^ are highlighted. All spectra were obtained in 10 mM phosphate buffer (pH 7) with 10% D_2_O.

Synthesizing variants confirmed the importance of the peptoid moieties, but NMR was necessary to identify which residues were directly coordinating the Cd^2+^. Cadmium is commonly used as a surrogate for other divalent cations when studying proteins by NMR, but few complexes have been characterized in order to probe small molecule ligand–Cd^2+^ interactions.^35^ Sequence 2 was selected for this analysis, as it was identified as the highest affinity sequence. An initial ^1^H-NMR spectrum of the peptoid alone (Fig. 3) had notably fewer rotamers than the tetramers we have previously studied.^17^ A 3 : 1 ratio of two distinct conformers was apparent instead of the overlapping signals of many rotamers. This conformational restriction could be due to the unique tertiary amines in this structure, but we propose that the two conformers result from the *cis* and *trans* states available to the d-proline.^36^ There have been many previous reports of the structure of peptoids with chiral centers in the side chains.^37^,^38^ However, an evaluation of the available rotamers has not been previously reported.

From ^1^H TOCSY (ESI, Fig. S6a†) and ^1^H–^13^C HMBC (ESI, Fig. S6b and S7a†) spectra, each of the relevant signals from Sequence 2 was assigned. The HSQC spectrum shown in Fig. 3 (full spectrum in ESI, Fig. S7b†) displays the behavior of Sequence 2 upon titration with Cd^2+^. With increasing equivalents of Cd^2+^ the methylene peaks of the Nthe residue (Fig. 3, signals A and B), in addition to those alpha to the N-terminus (Fig. 3, signal C), displayed distinct chemical shifts. The shifts observed in the protons in the Nthe residue are comparable to those observed for the methylene hydrogens in glutathione.^39^ However, the methylene hydrogens in glutathione display separate diastereotopic signals whereas the corresponding methylene hydrogens in Sequence 2 are identical. A possible binding mode supported by this observation is that the thiol and carboxylic acids coordinate the Cd^2+^ in a symmetrical fashion.

### Depletion of cadmium from biological media

The initial characterization confirmed the affinity of the identified structures for Cd^2+^. The next step was to confirm the selectivity of the ligands within the complex mixture of ions, small molecules, and proteins present in human serum. Since affinity does not necessarily correlate with selectivity, the four highest affinity peptoids that were repeatedly identified in the screening process (Sequences 1, 3, 9, and 14) and the highest affinity ligand (Sequence 2) were resynthesized on a Tentagel NH_2_ support compatible with both organic solvents and water (sequences are listed in Fig. 2b, and MALDI-TOF spectra are displayed in ESI Fig. S3†). Each of these ligands was exposed to three biological mixtures: (1) serum replacement, (2) serum replacement with the addition of relevant transition metals (100 μM: Fe^2+^, Mn^2+^, Zn^2+^), and (3) human serum from AB clotted whole blood. Each of these experiments contained an added cadmium concentration of 10 μM with a ten molar equivalent excess of the peptoid ligands on the resin.

**Fig. 4:**
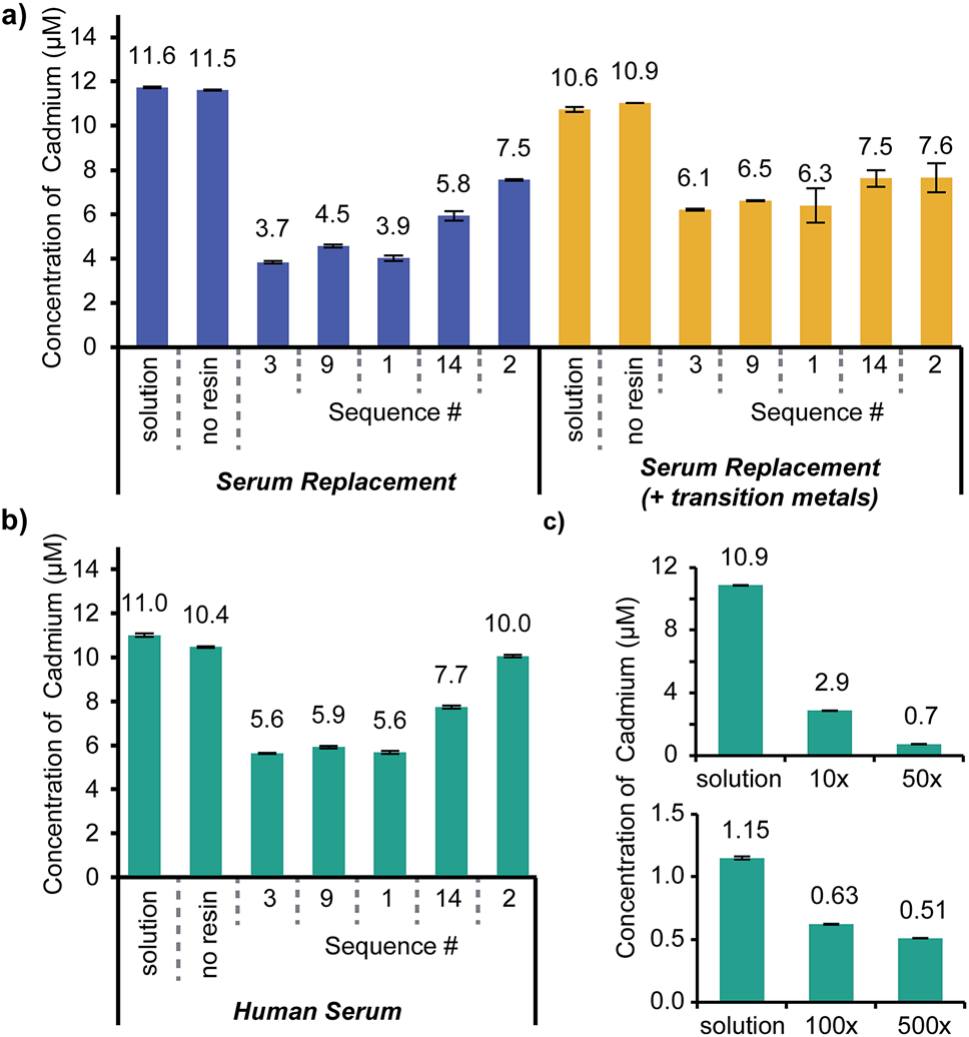
Evaluation of Cd^2+^ binding efficacy of peptoid ligands in human serum and mimetic solutions. (a) Serum replacement with and without the addition of biologically relevant transition metals (Fe^2+^, Mn^2+^, Zn^2+^, 100 μM) and (b) human serum were prepared with a toxic level of Cd^2+^ (10 μM) and exposed to five peptoid sequences attached to the resin (10 eq relative to Cd^2+^). The amount of cadmium remaining after a 24 h incubation was measured with ICP-OES. The cadmium concentration in the initial solution and a sample with no resin are reported, in addition to those exposed to ligands. (c) The depletion ability of Sequence 3 in human serum was additionally evaluated with more equivalents (X = eq relative to Cd^2+^) of the ligand and at a lower concentration of Cd^2+^ (1 μM). All error bars represent the standard errors of each sample set (*n* = 3).

Similar trends were apparent in each of the serum solutions evaluated (Fig. 4a and b). The most cadmium was depleted from the serum replacement alone, and most of the peptoids were comparably effective in the serum replacement with transition metals and the human serum, validating the selection of the former as a screening medium. Notably, Sequence 2, the ligand with the highest affinity for Cd^2+^, was outperformed by the other sequences in the serum replacement and human serum. Since the addition of the transition metals did not impact the efficacy of Sequence 2, it is likely that the binding was impeded by additional components of the serum, such as other ions or proteins. Sequence 14 deviated from the consensus exhibited by the other sequences evaluated (thiol–carboxylic acid–carboxylic acid–butyl) by including Npip in the middle of the structure; therefore, it is unsurprising that it was not as selective as the other ligands. Sequences 1, 3 and 9 appeared to perform equivalently in each medium, as might be expected by the minor differences in their structures.

**Fig. 5:**
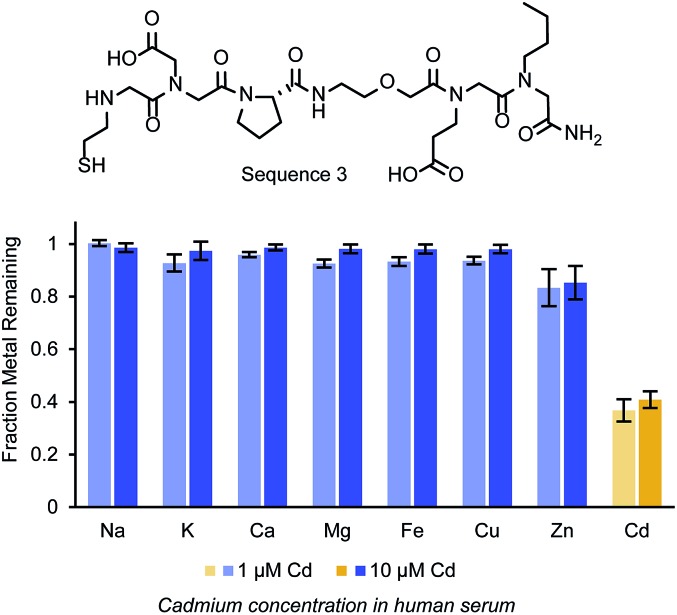
Evaluation of selectivity of peptoid ligands for Cd^2+^ in the presence of biological divalent cations in human serum. Two concentrations of cadmium (1 μM and 10 μM) were exposed to Sequence 3 (100 μM on resin) for 24 h, and the remaining concentrations of each of the divalent cations were measured using ICP-OES. All error bars represent the standard errors of each sample set (*n* = 5).

In chelation therapies, it is feasible and common to use large excesses of chelating agents. As one example, studies investigating EDTA as a chelation therapy used up to 500 equivalents (1.5 g kg^–1^) of the small molecule relative to the cadmium concentration in an *in vivo* study.^40^ To determine how effective the peptoids could be at higher molar ratios, Cd^2+^ was added to human serum at concentrations of 1 and 10 μM and exposed to various amounts of Sequence 3 on resin, as a representative structure (Fig. 4c). Sequence 3 (structure in Fig. 5b) was chosen as a representative ligand, but we anticipate similar results with Sequences 1 and 9. Penicillin and streptomycin were added to these serum samples to prevent the growth of bacteria, which could accumulate in the resin and block ligand access. In the 10 μM Cd^2+^ serum samples, 10 and 50 molar equivalents of Sequence 3 on resin were incubated with the samples and removed 73% and 94% respectively of the cadmium. More equivalents of the peptoid could not be added to these samples while still allowing full swelling of the resin. Therefore, to demonstrate further the depletion ability of the ligand, Sequence 3 was added to human serum samples with 1 μM Cd^2+^. The same quantities of resin containing Sequence 3 were added to these samples, and up to 56% of the Cd^2+^ was removed.

Although antibiotics were added to the samples to prevent bacteria from growing and accumulating in the resin used for the assay, it is still likely that larger proteins in the serum prevented the access of some ligands in the pores of the resin. Notably, even with the limitations of this assay, we were able to bring the cadmium concentration down to 0.51 μM—comparable to the limit for acute toxicity in whole blood reported by the Mayo Clinic (0.45 μM).^25^ If these ligands were integrated into dialysis systems that have been optimized for blood treatment, it is likely that blood cadmium concentration could be reduced to values well below this limit.

### Evaluation of ligand selectivity

Final experiments sought to evaluate the selectivity of the identified peptoid ligands more directly. To explore this, Sequence 3 was exposed to a solution with relevant ions (Fe^2+^, Mg^2+^, Mn^2+^, Ca^2+^, Zn^2+^, Cu^2+^, Ni^2+^, Cd^2+^ – approximately 10 μm) in a buffer compatible with each of these ions, bis–Tris (10 mM, pH 7.4). The samples were agitated with the beads using a nutator for 24 h at room temperature. The ligands were at a lower molar excess than in the serum assays (5 equiv instead of 10 equiv). In this solution, the ligands were still able to deplete the solution of over 70% of the Cd^2+^. As depicted in Fig. S8,† cadmium was the most significant ion depleted though the concentrations of the later transition metals were also lowered slightly.

These depletion selectivity experiments were next repeated in human serum using the native concentrations of the metal ions (Fig. 5). Serum samples with Cd^2+^ added at 1 and 10 μM were exposed to Sequence 3 on resin, and the remaining concentrations of Ca^2+^, Fe^2+^, Mg^2+^, Cu^2+^, Zn^2+^, and Cd^2+^ were measured. No significant change was measured for Mg^2+^, Ca^2+^, Cu^2+^, and Fe^2+^. Additionally, although the original concentration of Cu^2+^ and Zn^2+^ measured in the serum samples were close to 10 μM, much less was depleted from the serum samples than from the buffer samples. This is possibly due to native interactions of the ions with serum proteins and small molecules. Due to the chemical similarities between Cd^2+^ and Zn^2+^ (both being d^10^ metal ions) there are very few ligands with a selective affinity for cadmium.^41^ While a variety of small molecules have been developed as fluorescent sensors for cadmium, many are incompatible with water or have fluorescence variance due to factors other than specificity.^42^

## Conclusions

Through this work, we have demonstrated a combinatorial platform for the identification of readily-synthesized ligands capable of selectively chelating Cd^2+^ in a complex environment. Using the identified ligands, we demonstrated the capacity to remove cadmium from human serum samples with unprecedented performance. These molecules can be applied not only to the treatment of cadmium poisoning, but also in the ongoing investigations of the mechanisms of its toxicity. We are currently exploring the development of simple economical devices for the detection and treatment of cadmium poisoning by immobilizing these ligands on alternative supports. Additionally, we are continuing to explore the applications of this ligand identification platform in a variety of fields, including metal separations and actinide sequestration.

## Supplementary Material

Supplementary information
