# Supplementary material for: Development of peptoid-based ligands for the removal of cadmium from biological media
Source: Chem Sci. 2015 May 14;6(7):4042–8. doi: 10.1039/c5sc00676g (PMC4762606; doi:10.1039/c5sc00676g)
Supplement: Supplementary file 1 [file SC-006-C5SC00676G-s001.pdf]

Electronic Supplementary Material (ESI) for Chemical Science.
This journal is © The Royal Society of Chemistry 2015

Development of Peptoid-Based Ligands for the Selective Chelation of Cadmium in Biological Media

Abigail S. Knight,† Effie Y. Zhou,† Matthew B. Francis †,‡,*

†Department of Chemistry, University of California, Berkeley, CA, 94720, and ‡The Molecular Foundry at Lawrence Berkeley National Laboratory, Berkeley, CA, 94720

mbfrancis@berkeley.edu

Supporting Information

Table of Contents **Page**

S.1	General procedures and materials	S2
S.2	Instrumentation and sample analysis	S2
S.3	Library synthesis	S2
S.4	Screening to identify metal-binding sequences	S3
S.5	Metal ion removal and photocleavage	S3
S.6	Single bead sequencing	S4
S.7	Binding constant evaluation via UV-vis titration	S4
S.8	Cadmium depletion analysis with ICP-OES	S4
S.9	References	S5
S.10	Representative MALDI-TOF and MALDI-TOF MS sequencing spectra (Figure S1)	S6
S.11	Structures of unique peptoids identified in the cadmium screens (Figure S2)	S7
S.12	Representative MALDI-TOF spectra of resynthesized peptoids (Figure S3)	S8
S.13	Quantification of binding affinity of peptoid ligands for Cd^{2+} (Figure S4)	S9
S.14	Structures and binding curves for Sequence 2 variants and glutathione (Figure S5)	S10
S.15	NMR characterization of Sequence 2 (Figure S6)	S11
S.16	NMR characterization of Sequence 2 (Figure S7)	S12
S.17	Comparison of depletion of Cd^{2+} vs. biological cations (Figure S8)	S13

General Procedures and Materials

Tentagel MB NH_2 resin (140-170 μm, 0.3 mmol/g), used for library synthesis, was purchased from Rapp-Polymere (Tuebingen, Germany). Library synthesis steps were performed in fritted disposable chromatography columns (Bio-Rad, Hercules, CA). During the reactions, the resin suspensions were slowly rotated using a nutator (Fisher Scientific, USA). The photolinker (Fmoc-(*R*)-3-amino-3-(2-nitrophenyl) propionic acid) and Fmoc-6-aminohexanoic acid were purchased from Chem-Impex (Wood Dale, IL). The 1PEG ((2-(Fmoc-amino)ethoxy)acetic acid) and 2PEG ((2-(2-(Fmoc-amino)ethoxy)ethoxy)acetic acid) linkers were purchased from Iris Biotech GMBH (Marktredwitz, Germany). Water (dd-H_2O) used was deionized using a Barnstead NANOpure purification system (ThermoFisher, Waltham, MA). Serum replacement (KnockOut Serum Replacement) was purchased from Life Technologies (Carlsbad, CA) and human serum was obtained from AB clotted whole blood (Sigma, St Louis, MO). All other materials were purchased from commercial sources and used without further purification, except as noted below. Small scale centrifugation was performed in a Galaxy Mini Star (VWR, USA) and lyophilization was performed using a Labconco Freezone 4.5.

NMR Characterization

^1H and ^{13}C spectra were obtained on a Bruker Biospin (900 MHz) spectrometer. Peaks were calibrated using a DSS (4,4-dimethyl-4-silapentane-1-sulfonic acid) standard, DMSO (2.500, q), or H_2O (4.790, s) and spectra were analyzed using TopSpin software. For experiments in water, water was suppressed with excitation sculpting.[1]

Library Synthesis

Of the seven amine monomers, piperonylamine, butylamine, and histamine were used as purchased and incorporated without protecting groups. *N*-boc-ethylenediamine was purchased and used as received. Before use, the glycine and β-alanine monomers (purchased as hydrochloric acid salts with *t*-butyl ester protecting groups) were treated with 1 M NaOH and extracted into a 15:85 v/v isopropanol-chloroform mixture which was dried over sodium sulfate and concentrated, yielding the monomers as the free bases. Cysteamine was protected with a trityl group using a procedure reported by Maltese.[2] The ability of each amine to incorporate into a peptoid using the chemistry described below was previously verified by synthesizing pentamers with alternating benzylamine groups and test monomers.[3, 4] The library was synthesized on Tentagel MB NH_2 resin, which was initially swollen in dichloromethane (DCM). Standard Fmoc solid-phase synthesis (using HCTU as a coupling agent) was used to incorporate the three residues in the linker through amide bond formation. For the first variable position, the resin was split evenly into seven different fritted columns. Acylation and the addition of the first amine were performed according to the procedure developed by Zuckermann *et al.* for the incorporation of heterocyclic amines.[5] Acylation was achieved by gently agitating resin beads for 5 min with a solution of chloroacetic acid (6.8 eq, 0.4 mM) in dimethylformamide (DMF) and a solution of diisopropylcarbodiimide (8 eq, 2 M) in DMF. The acylation solution was removed via filtration and the resin beads were rinsed with DMF. Next, the amines were added as 2 M solutions in DMF. After 2-12 h of gentle agitation at room temperature, the resin samples were isolated via filtration and rinsed with DMF. All 7 resin aliquots were then pooled and mixed in DCM for 5 min. Subsequently, the resin was isolated via filtration, vacuum dried, and split into a new set of 7 fritted columns. The acylation and amination steps were repeated to add amines at the second variable position. The library was pooled and split into three separate containers for the addition of the turn sequences. To insert the linker and proline monomers into the peptoid backbone, solutions of the linker (20 eq, 0.6 mM in DMF) and diisopropylcarbodiimide (19 eq, 3.2 mM in DMF) were incubated with the resin for 30 min with gentle agitation. The proline was attached using standard solid-phase peptide synthesis (with HCTU as the coupling agent), after which the standard peptoid synthesis procedure was resumed

to couple the remaining amines. After the final amination, the resin pools were recombined and agitated gently in a 20% solution of 4-methylpiperidine in DMF to remove any acylation adducts on the imidazole groups. After isolation via filtration, the resin was rinsed with DMF and DCM, then dried under a vacuum. The protecting groups were removed by incubation with a cleavage solution (95:2.5:2.5) trifluoroacetic acid:water:triisopropylsilane) for 1.5 h. The resin was then rinsed with DCM, dried under vacuum, and stored, protected from light, at 4 °C until use.

Screening to Identify Selective Metal-Binding Sequences

To equilibrate the beads before the screen, a 50 mg portion of the resin (representing more than 3 times the size of the library to ensure inclusion of each sequence) was swelled in water (1 h). The library aliquot was then incubated with $CdCl_2$ (250 µM) in 20-40 mL of screening solution (2.5 mM in $ZnCl_2$, $MnCl_2$, and $FeCl_2$ in serum replacement) for 1 h. The aliquot was then rinsed three times by incubation with 1 mL water in order to facilitate removal of ions retained by non-specific binding.

For visualization and bead selection, the resin was transferred to a Petri dish. Approximately 500 µL of cadmium-specific dye, cadion, (0.23 mM cadion and 20 mM potassium hydroxide in ethanol) was applied, and the solvent was evaporated from the Petri dish under a gentle flow of nitrogen in order to trap the dye and the metal within the individual beads. The aliquot in the Petri dish was examined using a Leica S6D Microscope (Leica, Germany). The beads with the most intense colors were manually selected for ligand identification. All photographs were taken with and iPhone (model 4 or 5).

Metal Ion Removal and Photocleavage

The removal of the metal ions was found in previous work to be essential before interpretable MALDI-TOF MS sequencing data could be obtained. Beads selected from the library screen were deposited onto the membrane of a 0.5 mL 0.2 µm centrifugal filter unit (Millipore, Billerica, MA). A mixture of Amberlite cation (Na^+ form, 5 mg) and anion (Cl^- form, 5 mg) exchange resins was then added to the filtrate collection tube of the centrifugal filter unit. The filter and the collection tube were then filled with 1 M HCl. This setup was incubated with gentle agitation on a nutator for 2 h, allowing diffusion to occur through the 0.2 µm filter. The selected library members were isolated from the exchange resins and acidic solution by centrifugation of the filtration unit. Water (500 µL) was added to the filter unit, and the unit was gently agitated on a nutator for 15 min to rinse the library members. The water was then removed by centrifugation. This process was repeated twice to ensure removal of residual HCl.

The beads bearing hit sequences were removed from the filter unit and transferred to a Petri dish using ethanol (approx. 500 µL). The individual beads were captured using a pipet and placed into individual 0.6 mL Posi-Click tubes (Denville Scientific, South Plainfield, NJ). The volume in each tube was brought to 5 µL with absolute ethanol. To cleave the peptoids from single resin beads, the tubes were placed in a computer controlled ICH-2 photoreactor with UVA bulbs (Luzchem, Ottawa, Canada) for 8 h (various incubation times were screened). For mass verification of bulk peptoid samples, the beads were able to be directly cleaved with the nitrogen laser (337 nm) on the MALDI-TOF MS (Voyager-DE Pro, Applied Biosystems) in addition to the Nd:YAG laser (355 nm) on the MALDI-TOF-TOF MS/MS (4800 plus MALDI-TOF-TOF Analyzer, Applied Biosystems)

To determine whether unintended photoreactions were occurring with the unprotected N-terminal thiol, after one screen the library members were incubated in *N*-ethylmaleimide (25 µL, 10 mM, in dd-H_2O) for 30 min before photocleavage.

Single Bead Sequencing

After single-bead photocleavage, the ethanol was evaporated and replaced with 1:1 water:acetonitrile containing tris(2-carboxyethyl)phosphine (0.5 mM). The sample (0.5 μL) was mixed with 0.5 μL matrix solution (5 mg α-cyano-4-hydroxycinnamic acid in 1:1 water:acetonitrile with 0.1% trifluoroacetic acid and 0.6 M ammonium phosphate) and spotted directly on a stainless steel MALDI plate. MALDI-TOF MS (AB Sciex TF4800, Applied Biosystems) was used to identify the mass of each selected sequence, and MS/MS was used to fragment the peptoids for sequencing. Eighteen unique sequences were identified from this screening process.

Binding Constant Evaluation via UV-vis Titration

Each of the identified sequences was synthesized using the protocols described for library synthesis with Fmoc-Rink Amide MBHA resin (Anaspec, Fremont, CA) in place of the Tentagel MB NH_2 resin. The initial fluorenylmethyloxycarbonyl (Fmoc) group was removed with 20% 4-methylpiperidine in DMF. Following synthesis, a cleavage cocktail (95:2.5:2.5 trifluoroacetic acid:water:triisopropylsilane) was used to remove the peptoids from the resin, while also removing the protecting groups from the monomers. After evaporating the trifluoroacetic acid, the peptoids were precipitated from ether. The resulting precipitates were resuspended in water and purified using reversed phase chromatography on a semi-preparatory scale HPLC column (Agilent). The isolated fractions were concentrated using a speed vacuum (Labconoco, USA) and then lyophilized. The resulting sequences were stored dry at -20 °C before use in order to minimize disulfide formation. MALDI-TOF MS characterizations of the purified peptoids appear in **Figure S3**.

To evaluate binding constants for each hit sequence, $CdCl_2$ (0.3 - 5 mM) was titrated into a solution of peptoid (90 μL, 150 μM in 10 mM HEPES buffer, pH 7) in increments of 0.1-1 μL. The visible absorbance spectrum was measured at each increment using a Cary 50 spectrophotometer (Varian). The absorbance at the monitored wavelength (245 nm) was plotted against the concentration of cadmium and fit to a logistic binding curve using Origin software. The model used to fit the data is displayed in Equation 1, where A_1 and A_2 are the asymptotes of the data, p correlates to the slope of the curve, and x_0 is the inflection point used to approximate the dissociation constant.

$$y = (A_1 - A_2)/(1 + x/x_0)^p + A_2$$

Equation 1

Cadmium Depletion Analysis with ICP-OES

Five of the original eighteen hit sequences were carried forward to depletion experiments: S2 (which demonstrated the best binding constant from the UV-vis assay) and Sequence 1, Sequence 3, Sequence 9 and Sequence 14, which were all identified multiple times in the initial screens. These sequences were synthesized on Tentagel MB NH_2 (0.3 mmol/g) using the procedures described in the “library synthesis” section. A photolinker and two aminohexanoic acid residues were used to link the peptoid structures to the resin. The sequence identities were confirmed via MALDI-TOF MS by directly cleaving beads with the MALDI laser.

To evaluate these sequences’ ability to remove cadmium from serum replacement and human serum samples, several solutions were evaluated: serum replacement was artificially contaminated with either (1) $CdCl_2$ (10 μM) or (2) $CdCl_2$ (10 μM), $ZnCl_2$, $MnCl_2$, and $FeCl_2$ (100 μM); and human blood serum was contaminated with 10 μM $CdCl_2$. Three replicate samples of each of the five hit sequences were each

incubated for 24 h in the depletion solutions, such that the ligands were initially in ten-fold molar excess to the cadmium ions (assuming 100% modification of the resin). Following this incubation period, an aliquot from each sample was removed and diluted with nitric acid to a final concentration of 2% HNO_3; samples containing human blood serum were centrifuged (30 min, 4 ˚C, 4000 rpm) to aid in precipitation of denatured proteins. The concentration of cadmium remaining in those samples was determined by averaging five measurements of each sample with a Perkin Elmer 5300 DV optical emission ICP using scandium as an internal standard (Figure S4A-B).

Further depletion assays were carried out to determine the full potential of the peptoid ligands to deplete cadmium from human serum. Sequence 3 on resin was added to human blood serum with 10 μM cadmium (with 10-fold molar excess peptoid) and 1 μM cadmium (with 100-fold molar excess peptoid) (Figure S4C). In these samples penicillin (100 units mL^{-1}) and streptomycin (0.1 mg mL^{-1}) were added to prevent bacterial growth during the assays. This experiment was repeated to determine the selectivity of the binding, and the concentration of the biological cations (Na^+, K^+, Zn^{2+}, Mn^{2+}, Mg^{2+}, Fe^{2+}, Cu^{2+}, and Ca^{2+}) was measured in addition to cadmium. The Na^+ and K^+ ions were measured at 1:1000 dilution and the other ions were measured at a 1:10 dilution into nitric acid (final concentration 2%). One of the data points for the measurement of Na^+ and K^+ in untreated serum was determined to be a statistical outlier and was not included.

Finally, to evaluate selective binding of cadmium verses biological divalent cations at comparable concentrations, a solution of $CdCl_{2,}$ $ZnCl_2$, $MnCl_2$, and $FeCl_2$, $CuCl_2$, $Ni(OAc)_2$, $Mg(OAc)_2$, and calcium lactate (10 μM) and Bis-TRIS (10 mM, pH 7.4, BioXtra, Sigma-Aldrich (St Lous, MO)) was exposed to each of the resin-bound peptoids.

Supporting Information References

1. Hwang, T.; Shaka, A. J. Magn. Reson. Ser. A 1995, 112, 275–279.

2. Maltese, M. J. Org. Chem. 2001, 66, 7615–25.

3. Burkoth, T. S.; Fafarman, A. T.; Charych, D. H.; Connolly, M. D.; Zuckermann, R. N. J. Am. Chem. Soc. 2003, 125, 8841–8845.

4. Knight AS, Zhou EY, Pelton JG, Francis MB. J. Am. Chem. Soc. 2013, 135, 17488–17493.

5. Zuckermann, R. N.; Kerr, J. M.; Kent, S. B. H.; Moos, W. H. J. Am. Chem. Soc.1992, 114, 10646–10647.

a)

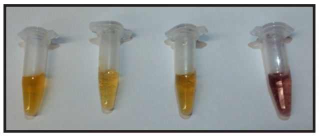

b)

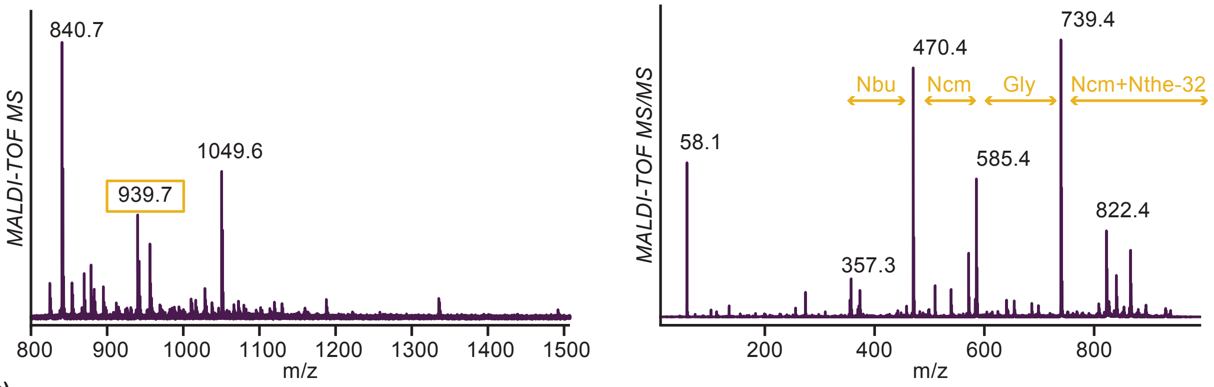

c)

Residue	Monomer Fragment Mass
Nce	129
Nbu	113
Nthe	117
Nae	100
Ncm	115
Nie	151
Npip	191

Residue	Mass of Turn plus Monomer Fragment	
	1PEG	2PEG
Nce	298	342
Nbu	311	355
Nthe	313	357
Nae	315	359
Ncm	327	371
Nie	349	393
Npip	389	433

d)

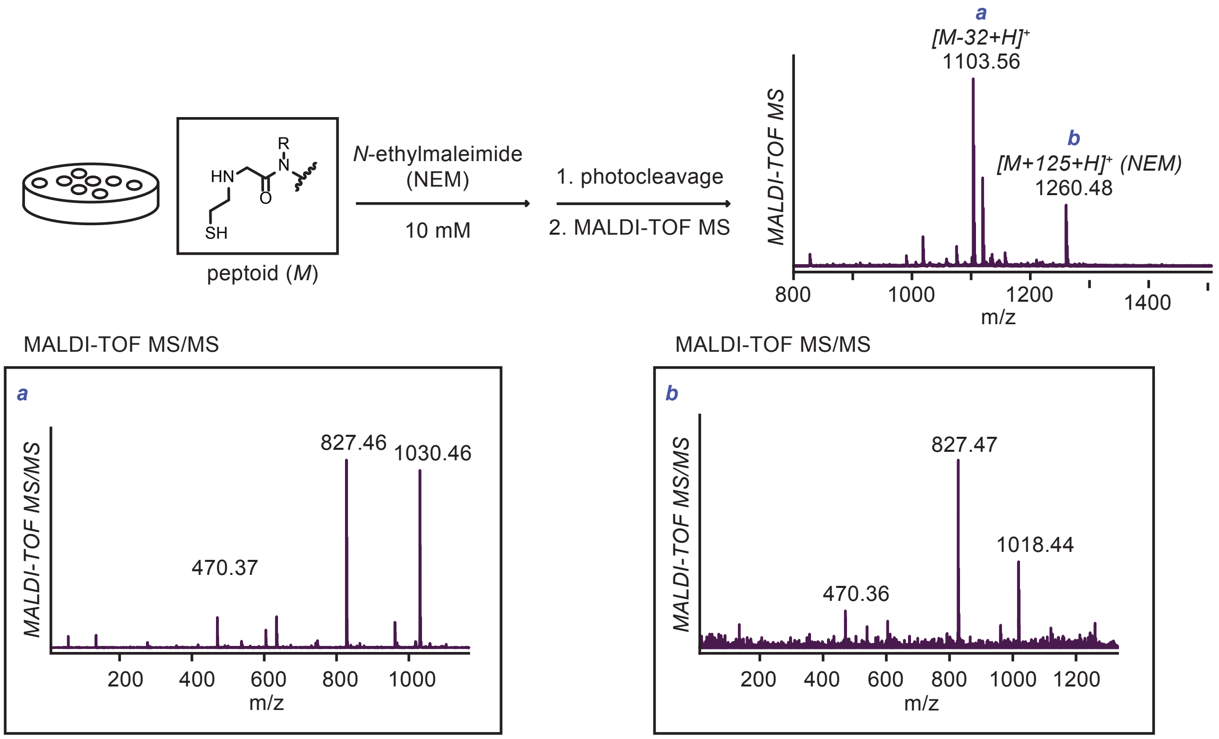

Figure S1. Representative data from library sequencing. a) Cadion dye added to relvant transition metals (4 mM). b) MALDI-TOF MS and MALDI-TOF MS/MS of Sequence 2 after screening and photocleavage. c) Table of masses of monomer fragments and the masses of the sum of the turn and the third monomer. The fragmentation that would lead to the intermediate is not visible due the decreased stability of the fragmented secondary amide verses a tertiary amide. d) Schematic of the *N*-ethylmaleimide (NEM) capping used to determine the mass of the peptoid before photocleavage. MALDI-TOF MS of an individual bead with both the NEM capped mass and the M-32 mass is shown in addition to the MS/MS spectrum for both masses.

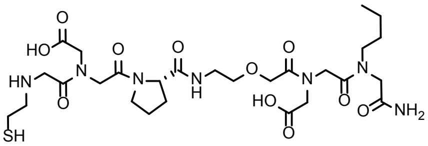

Sequence 1

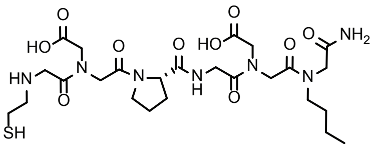

Sequence 2

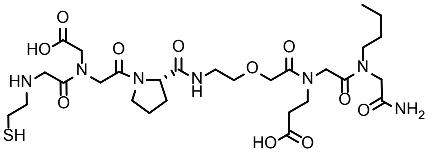

Sequence 3

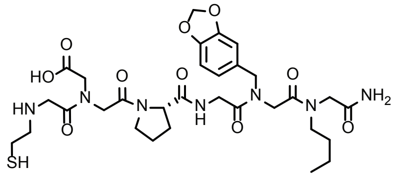

Sequence 7

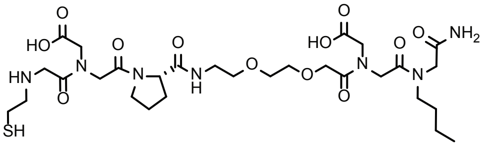

Sequence 9

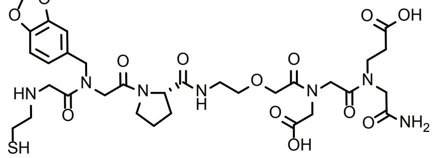

Sequence 14

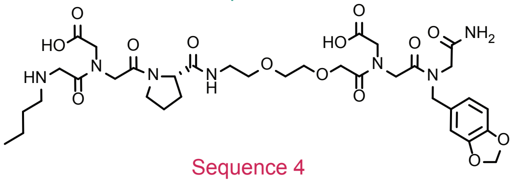

Sequence 4

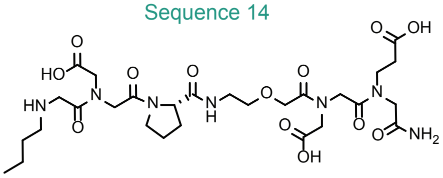

Sequence 5

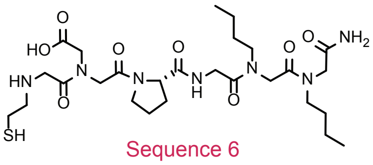

Sequence 6

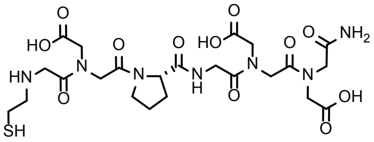

Sequence 8

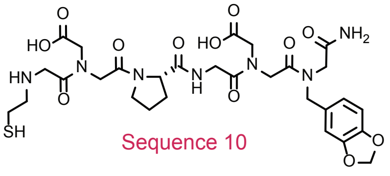

Sequence 10

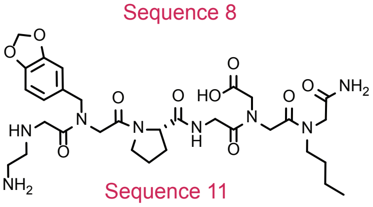

Sequence 11

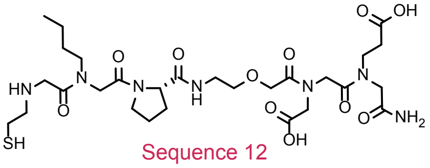

Sequence 12

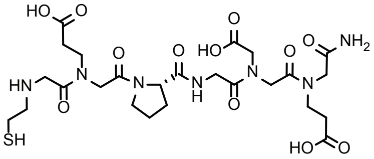

Sequence 13

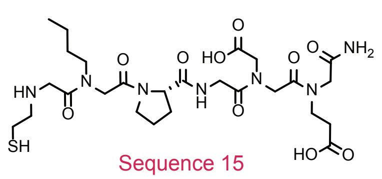

Sequence 15

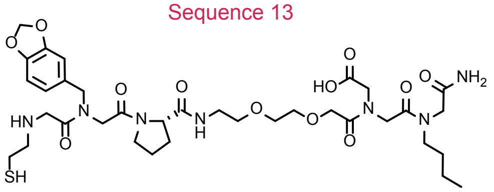

Sequence 16

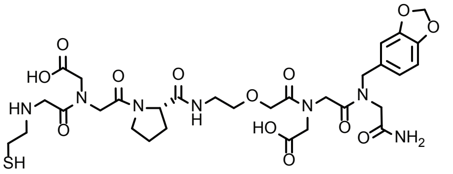

Sequence 17

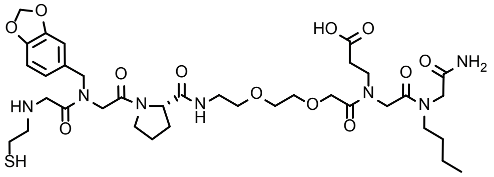

Sequence 18

Figure S2. Structures of each of the unique peptoids identified in the cadmium screens. The structures labeled in teal are highlighted in the main text.

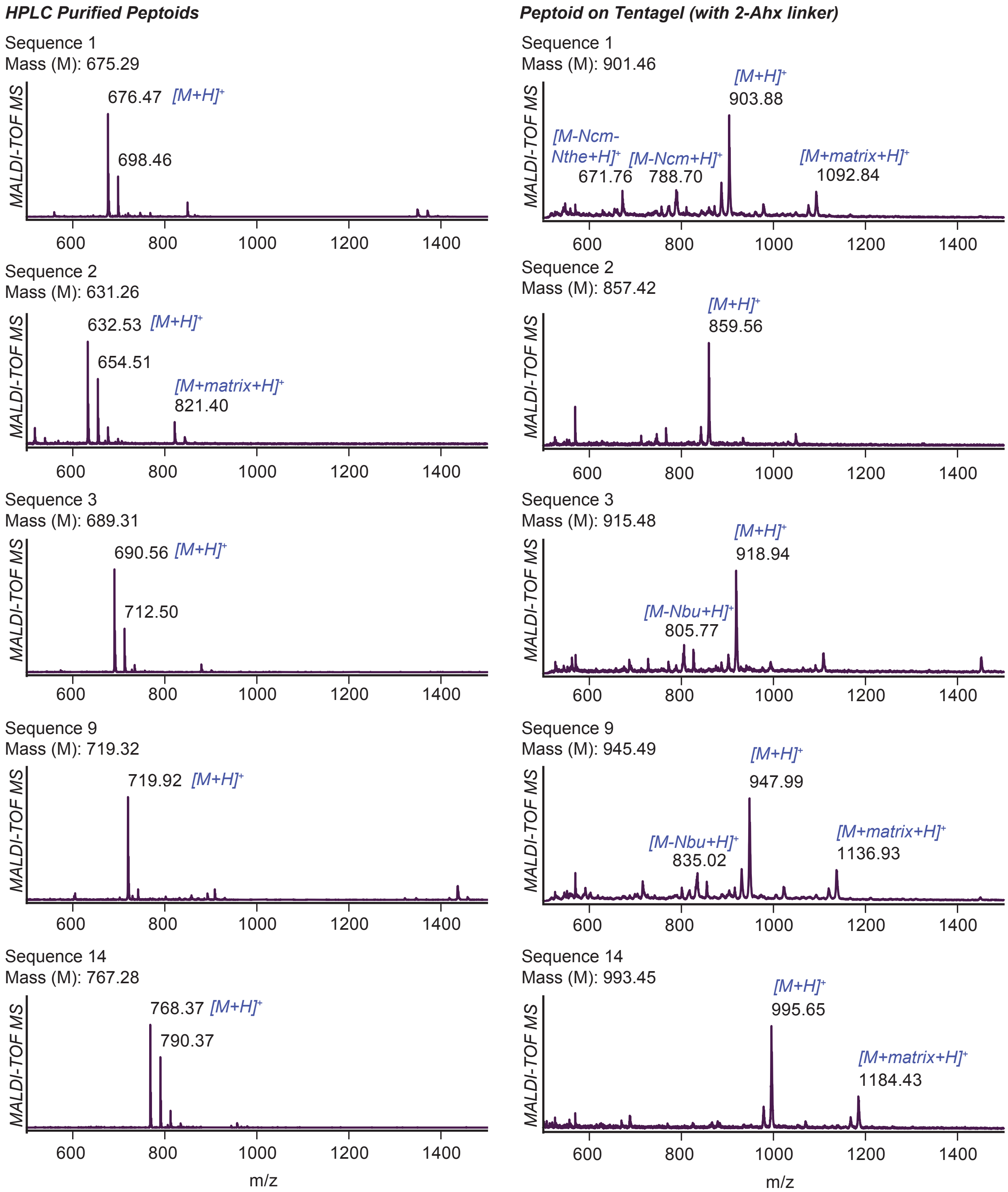

Figure S3. Representative MALDI-TOF MS spectra of HPLC purified peptoids and peptoids on Tentagel MB NH_2, directly cleaved with the MALDI-TOF MS laser. Corresponding structures are featured in Figure S2.

a)

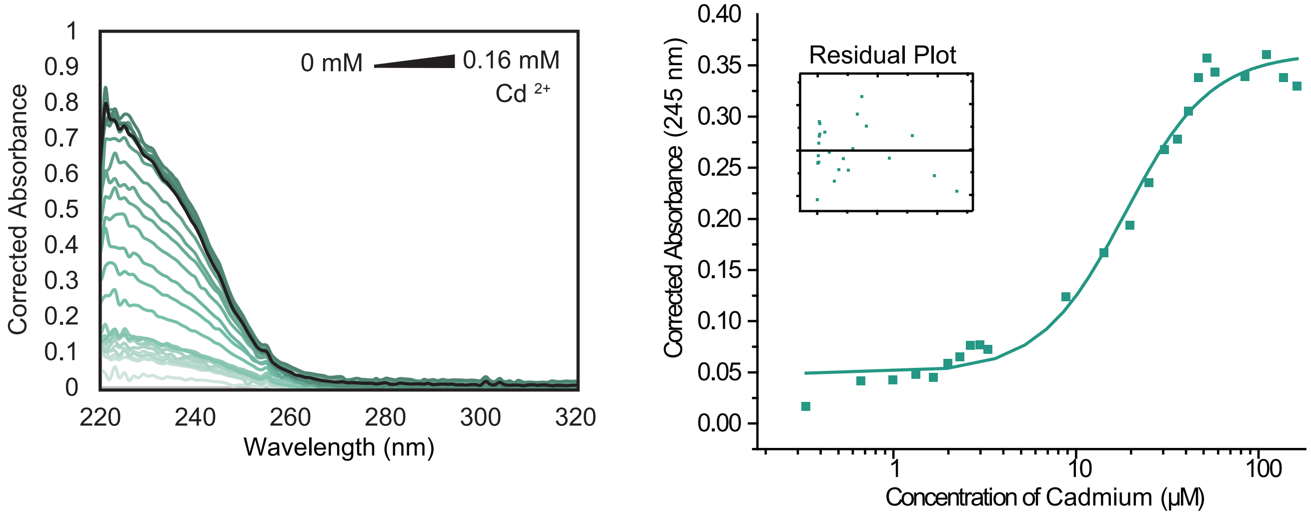

b)

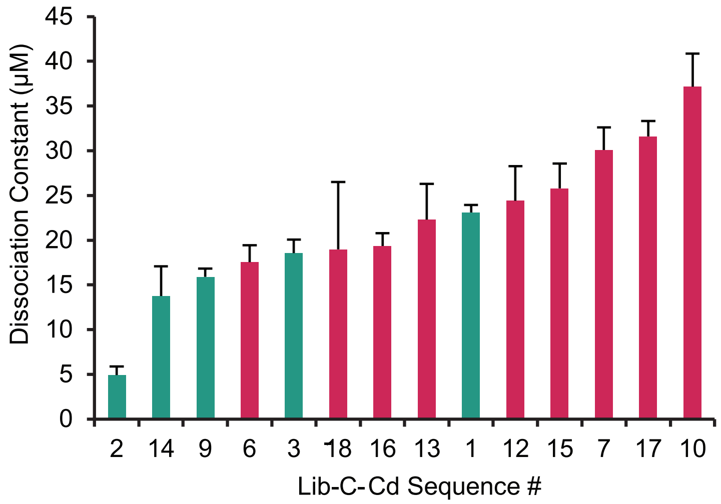

#	Replicates	K_d (μM)	Error	X^2	R^2
2		4.9	0.9	0.0001	0.963
14	2	13.7	3.3	0.0004	0.975
9	2	15.9	0.9	0.0002	0.988
6		17.5	1.9	0.0007	0.988
3	2	18.6	1.5	0.0003	0.981
18		19.0	7.5	0.0003	0.984
16		19.4	1.4	0.0018	0.978
13		22.3	4.0	0.0002	0.971
1	3	23.1	0.9	0.0001	0.990
12		24.4	3.9	0.0006	0.951
15		25.8	2.8	0.0003	0.987
7	3	30.1	2.5	0.0011	0.986
17		31.6	1.7	0.0003	0.988
10		37.2	3.7	0.0005	0.986

Figure S4. Quantification of binding affinity of peptoid ligands for Cd^{2+}. a) Example of a UV-vis titration of Cd^{2+} into a peptoid (Sequene 3, 150 μM) in HEPES (10 mM, pH 7). The curve was fit to a logistic function and the inflection point was used to approximate the K_d. The absorbance values have been normalized by subtracting the spectrum of the peptoid alone. b) Graph and table of the measured K_d values. Error bars represent the standard errors in the logistic fits.

a)

#	X1	X2	Turn	X3	X4
A	Ncm	**Nthe**	Gly	Ncm	Nbu
B	Ncm	Ncm	Gly	**Nthe**	Nbu
C	Nbu	Ncm	Gly	Ncm	**Nthe**
Y	Nthe	**Nbu**	Gly	Ncm	Nbu
Z	Nthe	Ncm	Gly	**Nbu**	Nbu

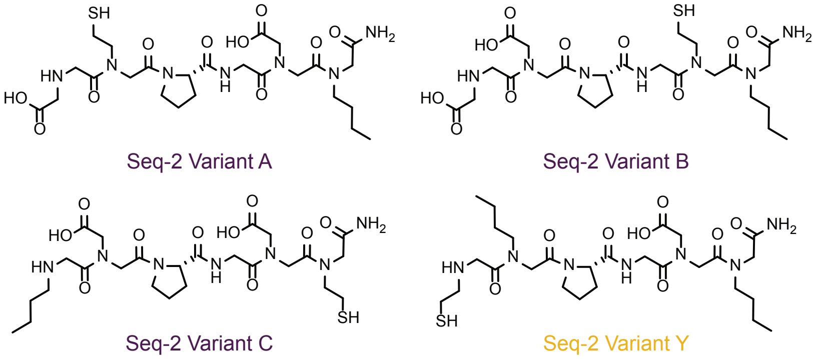

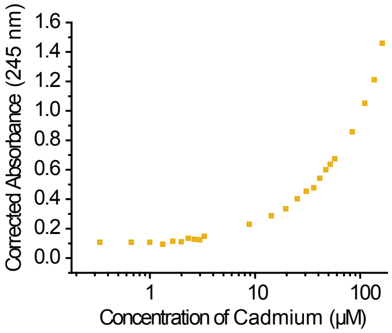

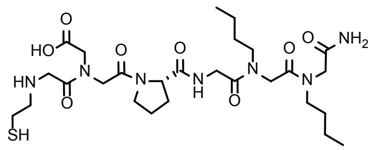

Seq-2 Variant Z

b)

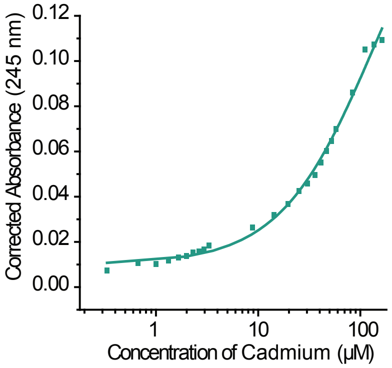

Glutathione

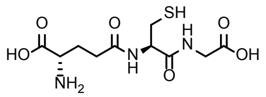

	K_d (µM)	Error	X^2	R^2
Glutathione	134	61	0.000008	0.993

c)

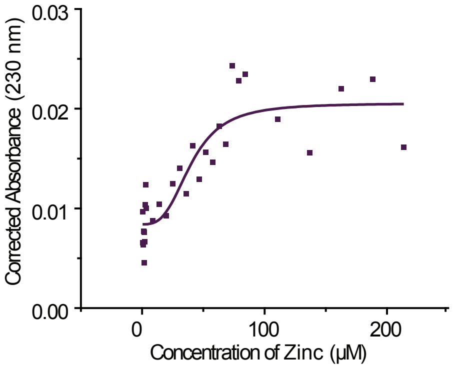

	K_d (µM)	Error	X^2	R^2
Zinc	40	7	0.000008	0.763

Figure S5. Stuctures and binding curves for Sequene 2 variants and glutathione. a) Structures of Sequence 2 variants and cadmium binding curve for Sequence 2 Variant Z, (150 µM) in HEPES (10 mM, pH 7). b) Structue and cadmium binding curve for glutathione (150 µM) in HEPES (10 mM, pH 7). c) Binding data from titration of Zn^{2+} into Sequence 2 (150 µM) in HEPES (10 mM, pH 7). The absorbance values have been normalized for all binding data by subtracting the spectrum of the peptoid alone.

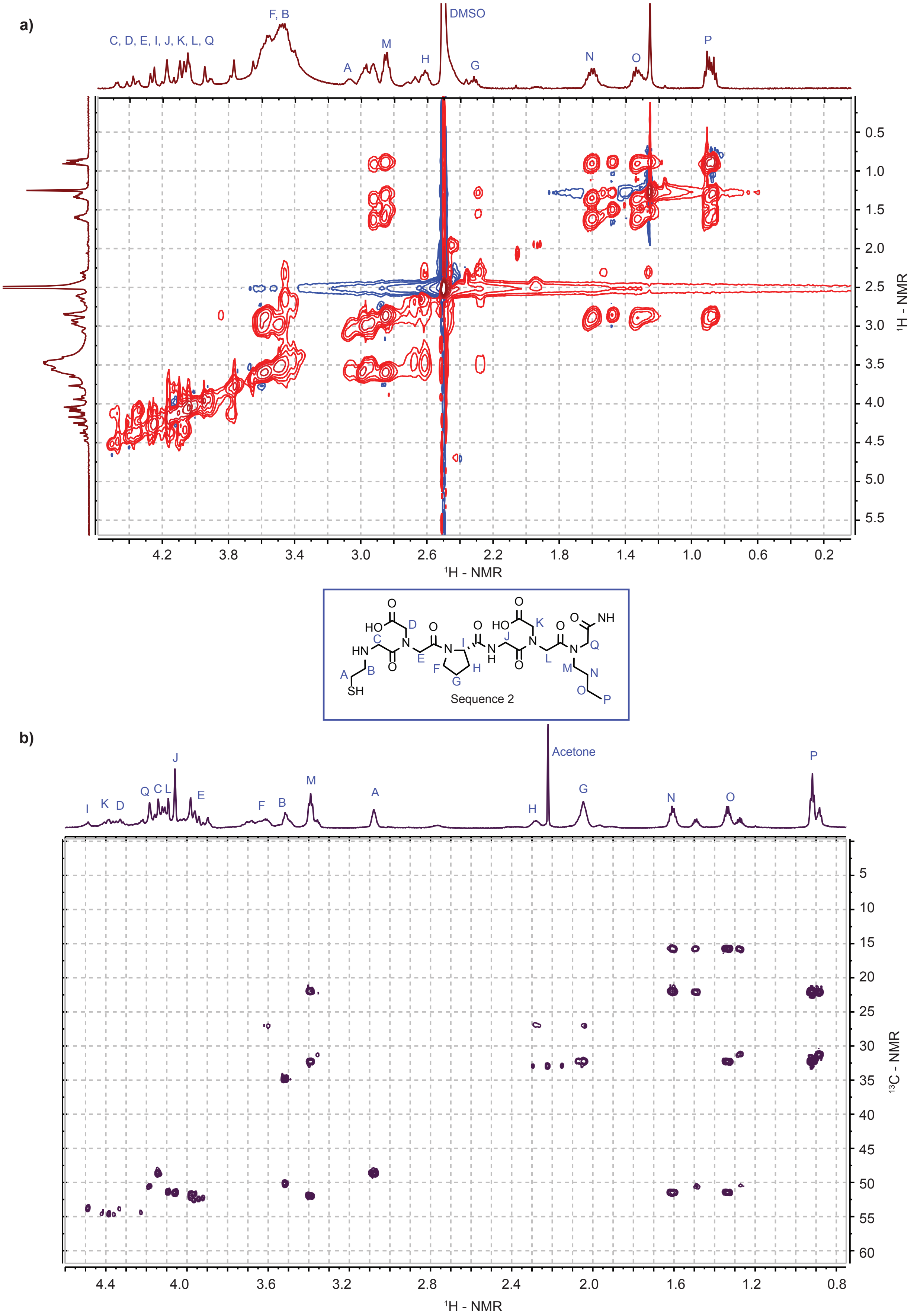

Figure S6. NMR characterization of Sequence 2. a) ^{1}H TOCSY (900 MHz) of Sequence 2. b) ^{1}H-^{13}C HMBC of Sequence 3 with cadmium (2 eq). All spectra were obtained in 10 mM phosphate buffer (pH 7) with 10% D_2O and 500 μM peptoid.

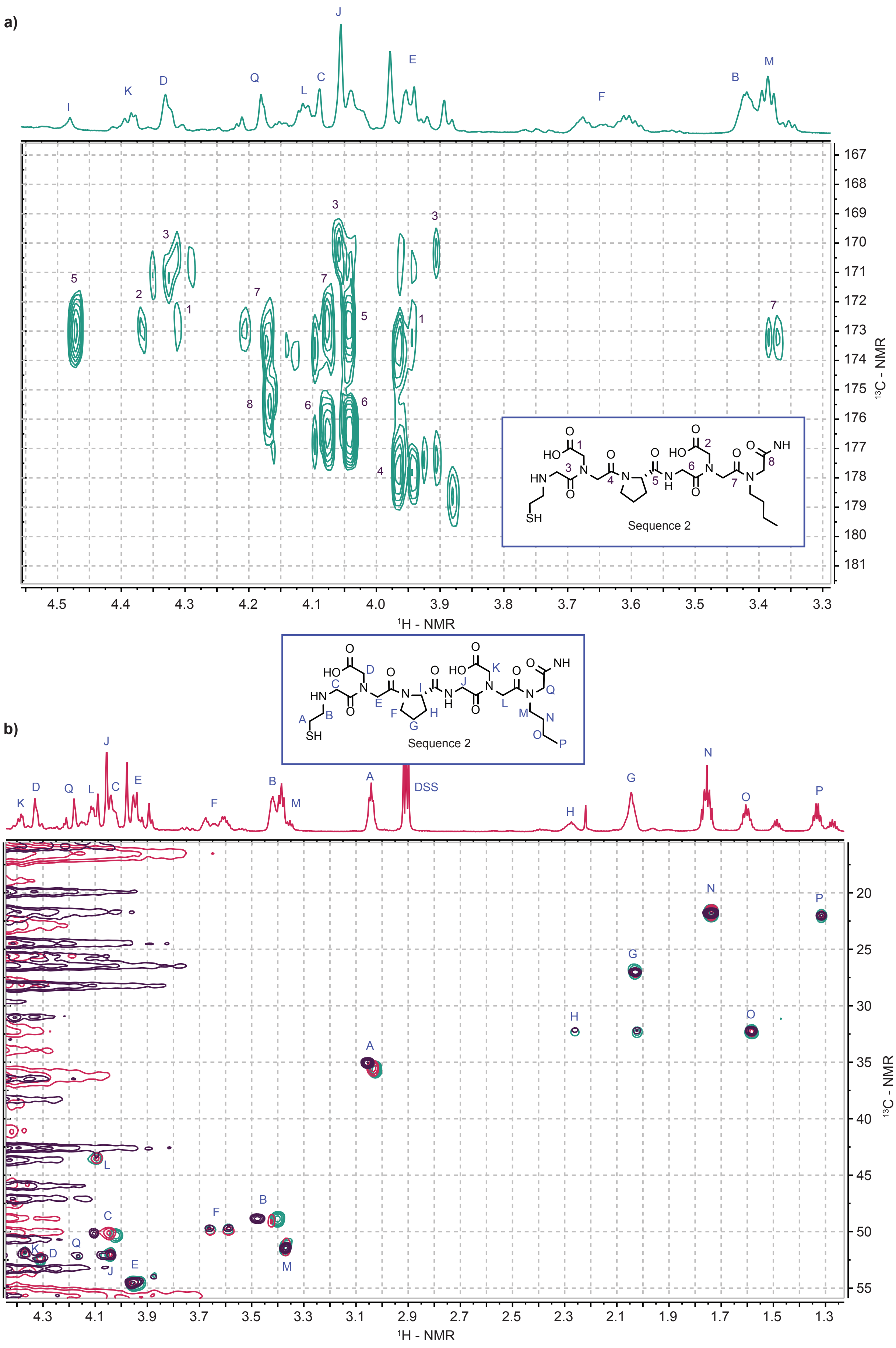

Figure S7. NMR characterization of Sequence 2. a) ^{1}H-^{13}C HMBC (900 MHz) of Sequence 2. b) ^{1}H-^{13}C HSQC of Sequence 2 without Cd^{2+} (teal), with 2 eq of Cd^{2+} (pink), and with 6 eq (purple).

a)

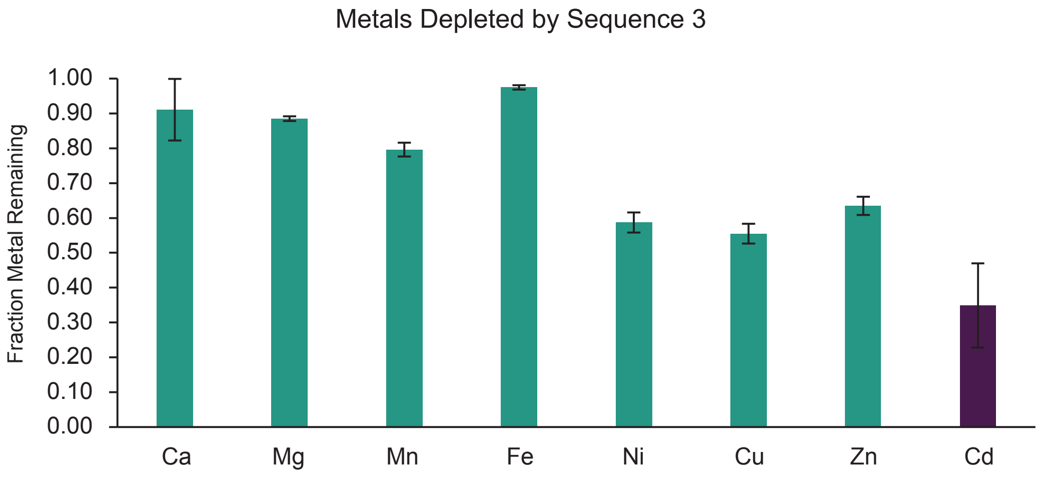

	Ca	Mg	Mn	Fe	Ni	Cu	Zn	Cd
starting solution (μM)	10.37	14.34	10.13	10.82	10.37	10.51	10.15	9.44
depleted soltuion (μM)	9.44	12.69	8.06	10.55	6.09	5.83	6.44	3.29

b)

Human serum with 1 μM Cd								
	K	Na	Fe	Zn	Ca	Mg	Cd	Cu
original solution (μM)	3812.59	14944.07	10.91	8.83	2250.09	144.31	0.64	11.53
Seq 3 depleted (μM)	3534.74	14991.00	10.19	7.36	2158.40	133.57	0.24	10.79
Human serum with 10 μM Cd								
	K	Na	Fe	Zn	Ca	Mg	Cd	Cu
original solution (μM)	3812.59	14944.07	10.91	8.83	2250.09	144.31	10.90	11.53
Seq 3 depleted (μM)	3710.21	14742.11	10.69	7.54	2219.71	141.56	4.46	11.30

Figure S8. Comparison of depletion of Cd^{2+} verses biological cations. a) Sequence 3 was exposed to each ion (Ca^{2+}, Fe^{2+}, K^{+}, Mn^{2+}, Mg^{2+}, Zn^{2+}, Cd^{2+}, Cu^{2+}, Ni^{2+}) at concentrations of approximately 10 μM and the remaining metal concentrations were measued with ICP-OES. The error bars represent the standard errors of each sample set (n=5). b) 1 μM and b) 10 μM ,were each exposed to two different amounts of Sequence 3 on resin (100 μM on resin and 500 μM on resin) for 24 h. The remaining concentrations of cadmium and serum cations were measured using ICP-OES.
